# Indication of a personality trait in dairy calves and its link to weight gain through automatically collected feeding behaviours

**DOI:** 10.1038/s41598-022-24076-x

**Published:** 2022-11-12

**Authors:** Charles Carslake, Francesca Occhiuto, Jorge A. Vázquez-Diosdado, Jasmeet Kaler

**Affiliations:** School of Veterinary Medicine and Science, University of Nottingham, Sutton Bonington Campus, Leicestershire, LE12 5RD UK

**Keywords:** Behavioural ecology, Animal behaviour

## Abstract

Farm animal personality traits are of interest since they can help predict individual variation in behaviour and productivity. However, personality traits are currently inferred using behavioural tests which are impractical outside of research settings. To meet the definition of a personality trait, between-individual differences in related behaviours must be temporally as well as contextually stable. In this study, we used data collected by computerised milk feeders from 76 calves over two contexts, pair housing and group housing, to test if between-individual differences in feeding rate and meal frequency meet the definition for a personality trait. Results show that between-individual differences in feeding rate and meal frequency were related, and, for each behaviour, between-individual differences were positively and significantly correlated across contexts. In addition, feeding rate and meal frequency were positively and significantly associated with weight gain. Together, these results indicate the existence of a personality trait which positions high meal frequency, fast drinking, fast growing calves at one end and low meal frequency, slow drinking, and slow growing calves at the other. Our results suggest that data already available on commercial farms could be harnessed to establish a personality trait.

## Introduction

In the same environment, conspecific animals often vary remarkably in their behaviour. Animal personality may contribute towards these differences^1^. Animal personality is the result of personality traits which are defined as underlying dispositions that drive temporally and contextually consistent between-individual differences in animal behaviour^2^. Personality traits have also been termed ‘personality axes’^3^ or ‘personality dimensions’^4^. Personality traits are of interest as they can help explain individual variation in life history and physiological traits^5,6^. For example, the ‘pace-of-life syndrome’ hypothesis predicts that individuals with consistently faster behaviours (e.g., more active) have higher metabolism, faster growth and earlier reproduction but suffer trade-offs such as a more easily compromised immune system and a shorter lifespan^7^. Indeed, there is evidence to suggest these trade-offs contribute towards the maintenance of differences in personality within a population^8^.

Whilst research into farm animal personality is in its infancy, studies indicate that personality traits, such as the boldness, sociality, and aggressiveness exist in livestock^9^. Personality traits cannot be measured directly because they are underlying (i.e., latent) traits^1,2^. Instead, personality traits must be inferred from measured behaviours. In livestock, personality traits are typically inferred by use of tests that measure behaviour, often under controlled conditions^4,10^. For example, numerous studies in cattle have used behavioural tests, such as exposure to a novel object, to measure related behaviours (e.g., latency to approach novel object, time in contact with novel object) to infer a personality trait (e.g., boldness)^11–13^. However, such tests are impractical beyond the research environment. A different approach is to collect repeated measures of behaviour and partition behaviour into between- and within- individual, by use of multi-level models^14,15^. These techniques allow between-individual variation to be quantified as a proportion of total variation (a measure termed repeatability)^16^. Behaviours that are more repeatable indicate consistent between-individual differences and are therefore particularly useful to the study of animal personality^17^. Where between-individual differences in behaviour are related, consistent over time, and consistent between contexts these differences indicate the presence of a personality trait^2^.

In dairy calves, recent studies have combined data gathered by computerised milk feeders with suites of behavioural tests to show that differences in calf personality are associated with differences in feeding behaviour and performance. For example, studies in calves have reported positive associations between the exploration personality trait, feed intake and weight gain^12,18^. However, despite the availability of suitable data gathered by precision livestock technologies, e.g.,^19–21^, few studies have attempted to harness these data to quantify between individual variation in behaviour directly (but see^22^). One example of this approach is from our recent work which indicates that substantial, temporally consistent between-individual differences exist for calf feeding behaviour, specifically for meal frequency and feeding rate^23^. Furthermore, behavioural types for feeding rate and for meal frequency were positively and significantly correlated (i.e., calves that had higher feeding rates visited the feeder more frequently whilst those with lower feeding rates visited the feeder less frequently) suggesting the presence of a personality trait which could be driving the reported differences. However, to meet the definition of a personality trait, between individual differences in associated behaviours must be contextually as well as temporally consistent^2^. To our knowledge, no studies have tested the contextual consistency of between-individual differences in calf feeding behaviour. Furthermore, since personality traits are associated with physiological and production differences, if between-individual differences in feeding behaviours are associated with a personality trait these differences may also help explain individual variation in weight gain. The present study examined between-individual differences of two distinct feeding behaviours, feeding rate and meal frequency, over two different contexts in 76 pre-weaned dairy calves. We chose to study feeding rate and meal frequency as these behaviours are mathematically distinct (i.e., the calculation of meal frequency is independent of feeding rate) and our previous research indicates that these behaviours are repeatable and related in pre-weaned calves^23^. The two contexts studied here were pair housing (where each pair had access to a milk feeding station) and group housing (where one milk feeding station was shared between sixteen calves). The objective of this study was to test if between-individual differences in calf feeding behaviour, as measured by a computerised milk feeder, indicate the presence of a personality trait.

## Materials and methods

### Data collection

#### Calf recruitment

The study was conducted at the Centre for Dairy Science and Innovation at the University of Nottingham, UK. 76 female Holstein Friesian calves were enrolled in the study between 21/06/2021 and 22/01/2022. The calves followed normal management procedures for the farm. Ethical permission was obtained for the School of Veterinary Medicine and Science, University of Nottingham (unique reference number 1481 150,603). All methods were performed in accordance with the relevant guidelines and regulations.

### Housing contexts

This study followed calves during the pre-weaning period (i.e., prior to any reduction in milk allowance). During this time, calves were managed in two different housing contexts. The first context was pair housing where the two calves closest in age were grouped together in a small (3 m × 2 m) straw bedded pen from birth. Each pair had continuous access to a feeding station (i.e., one teat for two calves). The second context was group housing where 16 calves closest in age were moved simultaneously from pair housing to a larger straw bedded group pen (6 m × 12 m). Pairs stayed with their conspecifics throughout. Calves were moved together once the youngest calf in the group of 16 was approximately 3 weeks old. During this second context, all 16 calves shared access to a single feeding station (i.e., there was one teat per 16 calves). The feeding stations’ dimensions were approximately 1 m  × 0.5 m. Each station was equipped with sides but there was no back gate to reduce competitive behaviour around the feeder.

### Feeding and colostrum

A more detailed description of the farm management and protocols can be found in Carslake et al. 2022^23^. For the first two days from birth, calves were fed colostrum followed by transition milk in line with farm protocols. From two days old calves transitioned onto milk replacer (Milkivit Energizer ECM, Trouw Nutrition GB) which was mixed by with warm water at 130 g/L and dispensed by the computerized feeder (Forster-Technik Compact Smart) via the teat present in the feeding station. Each calf was equipped with an RFID ear tag enabling the feeder to recognise its identity by use of an RFID reader and dispense a milk allowance. The total daily allowance was distributed evenly throughout the day and the maximum amount of milk dispensed within any 2-h period was limited to 2 L. The daily allowance started at 6 L at 2 days old and increased daily in line with age, reaching 8 L at 5 days old. From 8 days old, the total daily allowance increased more gradually, reaching a plateau of 10 L at 40 days old. During group housing, all calves, regardless of age, were fed 10 L daily for 35 days after which the allowance was reduced daily until reduced to zero after 60 days in the group pen. Calves had ad libitum access to concentrates (FiMLAC Sweet Start Pellets), chopped straw and water throughout.

### Data acquisition and selection

The computerized milk feeder used in this study logged feeding behaviour (calf identity, date, time start visit, time stop visit, milk consumption, drinking speed) during each visit a calf makes to the feeder. A new visit (row) was created whenever the RFID reader loses and then regains contact with a RFID tag. Data from the computerized milk feeder for the pair housed and the group-housed calves were downloaded and appended. From the pair housing we only included data where calves were a minimum of 7 days old since the daily increase in daily milk allowance was more gradual from this age (63mls/day). For each calf, we included 10 days of data. In the group housing period, the first week of data were excluded to allow a period of acclimatisation to the new pen and the subsequent 10 days were included in our analysis to ensure that an equal number of observations for each context. All calves in the group housing were on a level feeding plane of 10 L daily. We excluded any calves that were detected as sick during the data collection periods. Sick calves were detected during twice weekly health scores (Wisconsin score >  = 5; n = 20) or during visual twice daily observations by farm staff)—(see Table 1 for calves excluded).

**Table 1:** Number of calves included in analysis per cohort as per the inclusion criteria detailed in the methods and age of calves at start of data collection.

	Cohort 1	Cohort 2	Cohort 3	Cohort 4	Cohort 5	Cohort 6	Overall
**Calves**							
Calves excluded	7	0	0	5	2	6	20
Calves included	9	16	16	11	14	10	76
**Age at start of pair housing data collection (days)—included calves only**							
Mean	16	23	12	12	12	11	15
Min	7	7	7	7	7	7	7
Max	33	42	17	16	16	17	42
**Age at start of group housing data collection period (days)—Included calves only**							
Mean	32	40	40	43	28	29	35
Min	23	26	26	37	24	24	23
Max	39	54	50	49	36	35	54

### Ethical approval

Ethical permission was obtained for the School of Veterinary Medicine and Science, University of Nottingham (unique reference number 1481 150,603). All methods are reported in accordance with the ARRIVE guidelines^45^.

## Data processing

### Meal based criterion

A meal criterion was used to group visits by the individual that were close time into the same meal^24^. In this study we used a meal criterion of 100 s for both the pair and the group housing periods as detailed in Carslake et al. (2022)^23^ (also see supplementary material). Therefore, if two visits to the feeder by the same calf were separated by 100 s or less, they were grouped into the same meal. Those visits to the feeder by the same calf that separated by more 100 s were grouped into separate meals.

### Feeding behaviours

For each calf and for each day of the group-housing period, we calculated feeding rate and meal frequency to describe the calves’ feeding behaviour. These are detailed in Table 2.

**Table 2:** Definitions of feeding behaviours used.

Feeding behaviour	Definition
Meal frequency (number per day)	Daily sum of all meals. This variable includes meals where the calf is entitled to a milk feed and meals where the calf is not entitled to a milk feed
Feeding rate (ml/min)	Mean daily feeding rate. The feeding rate for each visit where the calf is entitled to a milk feed and consumes milk is calculated by the feeder. From this, we calculated the mean feeding rate

### Weight data

Birth weight for each calf was manually recorded by use of an electronic weight scale prior to colostrum feeding. During the group housing period, weight data was continuously collected by use of a partial weigh scale which was attached to the front of the automatic feeder. The partial scales collected a recording every time the calf visited the feeder. The partial weigh scale had been previously validated (personal correspondence from manufacturer). Weight data was downloaded from the feeder for group housing context, processed (see supplementary material) and weight at 70 days old was calculated. We chose weight gain between birth and 70 days old to represent weight gain for this study as 35 days was the average age at which calves moved to the group pen and 70 days therefore reflects weight gain across both contexts. Weight gain between birth and 70 days was calculated by subtracting birth weight from weight at 70 days old.

## Statistical analysis

We quantified behavioural variation and tested for relationships within and between contexts using a multivariate multilevel linear model. A multivariate model was also used to test for a relationship between feeding behaviour in the pair housing context and weight gain. Multivariate approaches were chosen for this study as these models carry forward the uncertainty around point estimates into correlations between them thereby generating estimates with valid estimates of uncertainty^25^. This is important since failing to account for uncertainty around point estimates (for example around estimates for individuals’ average behaviour or behavioural type) by simply carrying forward central estimates from individual linear mixed models into a further analysis, can lead to spurious p values when correlations are computed between these^25^. All statistical analyses were carried out using R software v3.5.1^26^. Code for the figures was adapted from Hertel et al. (2020)^21^.

### Multivariate model to quantify behavioral variation within and across contexts

We ran a multi-level multivariate linear model with repeated measures of each feeding behaviour in each context (feeding rate pair housing, meal frequency pair housing, feeding rate group housing and meal frequency group housing) as response variables. We used the “brms” package in R^27^. The model can be written as per Eq. (1).

1 $${\varvec{Y}}_{i} = \user2{X\beta } + \user2{Z\alpha } + {\varvec{\varepsilon}}$$

In the model $\alpha$ represents individual-specific random effect variation, $Y_{i} \user2{ }$ represents the four response variables, $X$ represents the fixed effects, *Z* the random effects. We controlled for the effects of age and birthweight by including them as fixed effects for feeding rate and meal frequency in the pair housing period. For the group housing we included age at grouping, number of days since grouping and their interaction term as well as weight at grouping as fixed effects. For all four response variables, we included individual Calf ID and cohort as random effects.

All distributions were specified as Gaussian. To capture a gaussian posterior distribution we log transformed the variables meal frequency pair housing and meal frequency group housing and boxcox transformed feeding rate pair housing and feeding rate group housing. All variables were scaled after transformation (mean = 0; SD = 1). We used uninformative priors for both fixed and random effects. We ran four chains for 12,000 iterations, a warmup of 4,000 iterations and a thinning interval of 4. Model diagnostics indicated satisfactory convergence with R̂ < 1.01 and effective sample sizes > 400. Posterior predictive checks indicated that the underlying Gaussian distribution was satisfactorily captured.

### Quantifying individual differences in feeding behaviors

Each calf had an individual specific intercept. For each response variable in our model, we calculated repeatability which represents the proportion of total variance that is explained by consistent differences between individuals. $Rpt$ was defined according to the equation below:

$$Rpt = \frac{{V_{{ind_{0} }} }}{{(V_{{ind_{0} }} + V_{eo} )}}$$

where $V_{{ind_{0} }}$ denotes the variance explained by differences between individual calves and $V_{eo}$ denotes the residual (within individual) variance.

$Rpt$ values are between 0 and 1. Higher values of $Rpt$ for a behaviour indicate the population is composed of individuals that behave consistently differently from each other whereas low values indicate that individuals are more similar. We describe our values for repeatability as high, moderate, or low in relation to a meta-analysis which summarized 759 estimates of repeatability from 114 studies and indicated that the mean level of repeatability was 0.37 [CI: 0.36–0.38]^17^. Here we describe values of repeatability from 0 to 0.25 as low, those from 0.25 to 0.50 as moderate, those above 0.5 as high.

### Correlations within and between contexts

Our multivariate model estimates the correlations and credibility intervals between each of the response variables at Calf ID level. This allows us to test for correlations between the adjusted average behaviours (i.e., the behavioural types) for meal frequency and feeding rate within each context as well as test for correlations between contexts. Statistically, a calf’s behavioural type corresponds to the individual level intercept from random intercept model for that behaviour.

To test if meal frequency behavioural types were correlated between contexts, we extracted the between-individual correlation and credibility interval for meal frequency in the pair housing and meal frequency in the group housing. To test if feeding rate behavioural types were correlated between contexts, we extracted the between-individual correlation and credibility interval for feeding rate in the pair housing and feeding rate in the group housing. To test if behavioural types for feeding rate and meal frequency are related within each context, we extracted the mean between-individual correlation and credibility interval for meal frequency and feeding rate in the pair housing context and the between-individual correlation and credibility interval for meal frequency and feeding rate in the group housing context. We describe the absolute values of correlation coefficients from 0 to 0.4 as weak, from 0.4 to 0.7 as moderate and above 0.7 as strong ^28^.

### Relationships between behavioral type and weight gain

We ran a second multivariate model to test if behavioural types from the pair housing period were associated with weight gain. This model had three response variables: weight gain between birth and 70 days, meal frequency in the pair housing and feeding rate in the pair housing. The model can also be written as per Eq. (1). In this model $\alpha$ represents individual-specific random effect variation, $Y_{i} \user2{ }$ represents the response variables, $X$ represents the fixed effects, *Z* the random effects. For the response variables feeding rate and meal frequency, fixed effects included age and birthweight. For the weight gain response variable fixed effects included birthweight only. For all three response variables, we included individual Calf ID and cohort as random effects. Since we only had a single observation per calf for the weight gain response variable, we fixed its residual variance to 0.002 in our prior specification^25^.

## Results

### Repeatability estimates by context

Repeatability estimates are reported in Table 3. In both contexts and for both behaviours, the degree to which individuals differed from each other as a proportion of total variation, as quantified by repeatability estimates, were moderate. These results imply that substantial between-individual differences exist for meal frequency and feeding rate in both the pair and group housing contexts.

**Table 3:** Mean, median, inter-quartile range, repeatability, and coefficient of variation in predictability for meal frequency and feeding rate for the same calves by context (pair housing and group housing). IQR and CI correspond to interquartile range and credibility interval respectively.

	Pair housing		Group housing	
	Feeding rate (ml/min)	Meal frequency (number per day)	Feeding Rate (ml/min)	Meal frequency (number per day)
Mean	609	18	836	10
Median	621	15	840	9
IQR	487–735	10–22	775–902	6–12
**Repeatability**				
Estimates	0.48	0.49	0.46	0.32
CI	0.39–0.58	0.29–0.49	0.37–0.56	0.22–0.41

### Correlations between contexts

**Figure 1:**
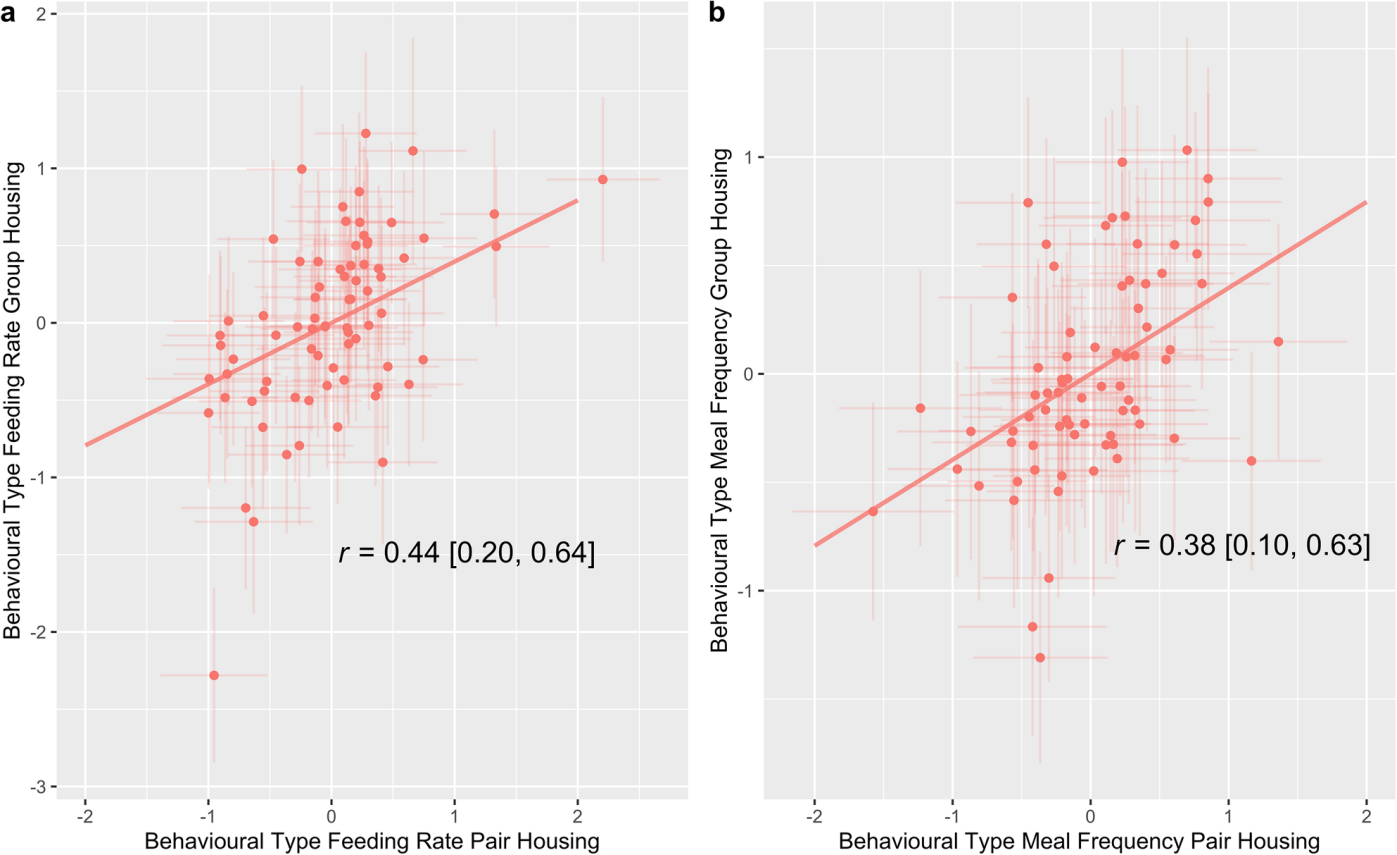
Visual representation of the line of best fit of the individual correlation (r) for feeding rate and meal frequency behavioural types between contexts. The credibility intervals are shown in square brackets. Panel (**a**) reports the correlation for feeding rate behavioural types between contexts. Panel (**b**) reports the correlation for meal frequency behavioural types between contexts. Posterior means and 95% credible intervals of point estimates for behavioural types are shown. The values on the x and y axes represent estimates for behavioural types which were calculated from the scaled response variables in the model.

For feeding rate, the correlation coefficient for individual calves’ behavioural types in the pair housing with individual calves’ behavioural types in the group housing was 0.44 (CI: 0.20 – 0.64). For meal frequency, the correlation coefficient for individual calves’ behavioural types in the pair housing with those from the group housing was 0.38 (CI: 0.10–0.63). These correlations are reported in Fig. 1.

Calves’ feeding rate in the pair housing context was positively and significantly correlated with feeding rate in the group housing context and calves’ meal frequency in the pair housing context was positively and significantly correlated their meal frequency in the group housing context. These results show that between-individual differences in feeding rate and meal frequency were positively and significantly correlated between contexts.

### Correlations within contexts

**Figure 2:**
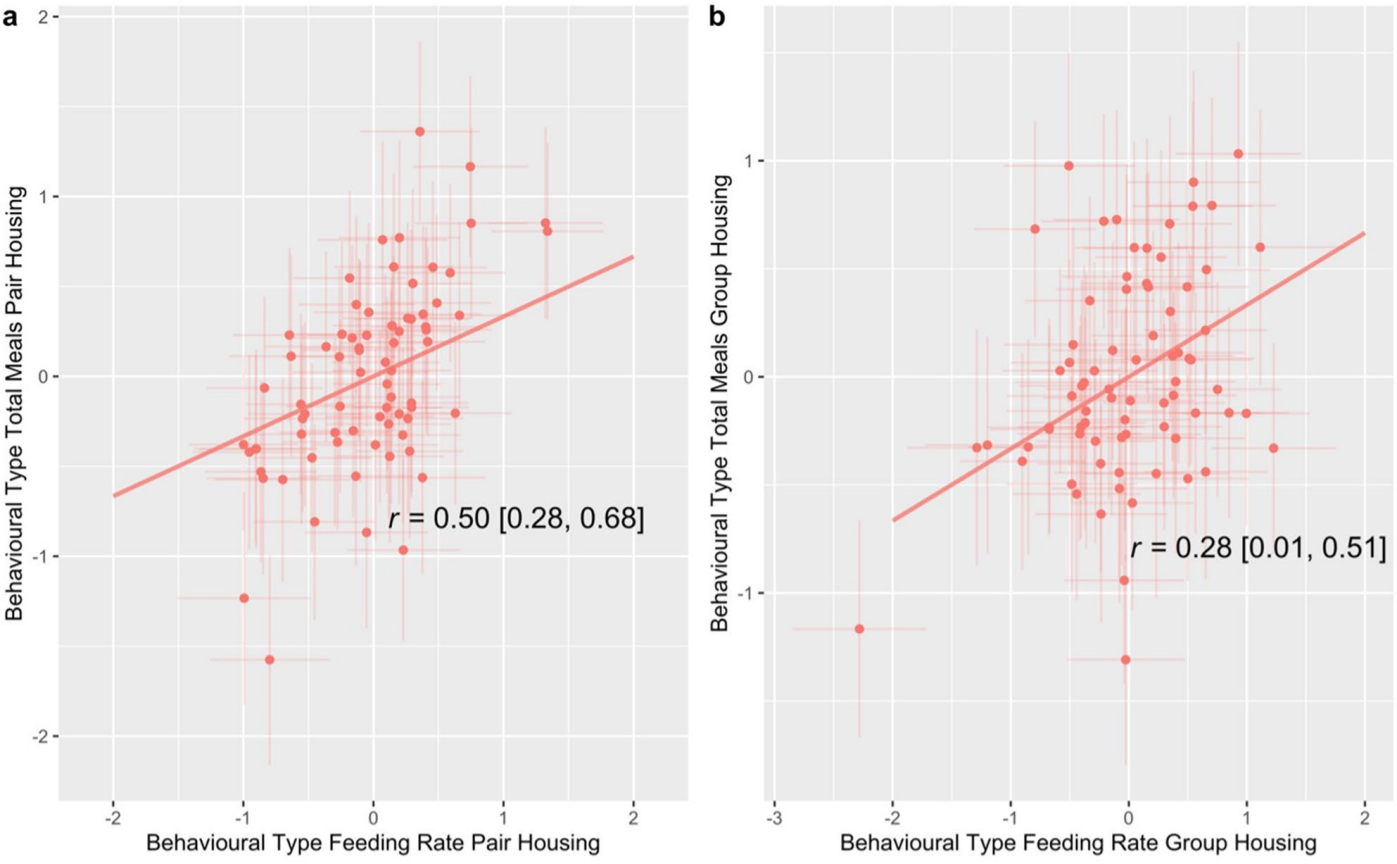
Visual representation of the line of best fit of the individual correlation (r) for feeding rate and meal frequency behavioural types within contexts. The credibility intervals are shown in square brackets. Panel (**a**) reports the correlation for feeding rate and meal frequency behavioural types in the pair housing and panel (**b**) reports the correlation for feeding rate and meal frequency behavioural types in the group housing. Posterior means and 95% credible intervals of estimates behavioural types are shown. The values on the x and y axes represent estimates for behavioural types which were calculated from the scaled response variables in the model.

The correlation coefficient between individual calves’ behavioural types for meal frequency in the pair housing and feeding rate in the pair housing was 0.50 (CI: 0.28–0.68). The correlation coefficient between individual calves’ the feeding rate behavioural types in group housing and the meal frequency behavioural types in the group housing was 0.28 (CI: 0.01–0.51). These correlations are reported in Fig. 2.

These results show that, within each context, between-individual differences are positively and significantly correlated at the intra-individual level (i.e., calves that had higher feeding rates tended to have higher meal frequency whilst those with lower feeding rates tended to have lower meal frequency).

### Correlations with weight gain

Results from the multivariate model which included weight gain alongside feeding rate and meal frequency in the pair housing indicate that the calves’ behavioural types for these behaviours were significantly and positively associated with weight gain. Correlation estimates were 0.42 (CI: 0.26–0.63) and 0.32 (CI: 0.12–0.52) for feeding rate and meal frequency respectively. This result shows that calves that had higher meal frequency and/or feeding rate in the pair housing had greater weight gain.

## Discussion

This study is amongst the first in livestock to use automatically collected data to test if between-individual differences in behaviour are consistent with the existence of a personality trait. Our results show that for meal frequency and feeding rate in calves:

I. Repeatability estimates were moderate and between-individual differences were positively and significantly correlated across contexts, indicating that substantial, temporally, and contextually stable between-individual differences exist.
II. Within each context, between-individual differences were positively and significantly correlated at the intra-individual level indicating that the expression of meal frequency and feeding rate are related.
III. Between-individual differences in young calves were positively and significantly correlated with future weight gain.

Together, these results support our proposal that a personality trait exists which is driving contextually and temporally consistent between individual differences in calf feeding rate and meal frequency. The proposed personality trait situates calves on an underlying axis with faster drinking, high meal frequency calves at one end and slower drinking, low meal frequency calves at the other. This approach offers substantial practical advantages compared to traditional behavioural tests and indicates that data which is already available on many commercial farms could be used to automatically phenotype calves.

Between contexts, individual differences in feeding rate and meal frequency were positively and significantly correlated. This study is the first to report contextual consistency of between-individual differences in feeding behaviour non-laboratory animals. It is worth highlighting that the physical and social environment between the two contexts (e.g., group size, access to the feeder etc.,) deviated substantially. For example, there was one feed station for every two calves in the pair housing context whereas in the group housing context, a single feed station was shared between sixteen calves. It is reasonable to assume that the calves were required to adjust their behavioural strategy to each context. Indeed, the mean meal frequency decreased from 17 in the pair housing to 11 in group housing, suggesting reduced access to the feed station in the group housing which could be due to increased competition around the feeder^29^. It is therefore particularly striking that, despite the substantial differences between contexts, between-individual differences were positively and significantly correlated between contexts indicating that the calves were highly motivated to display a preferred meal frequency and feeding rate. Whilst the correlations between contexts for feeding rate and meal frequency were weak to moderate (r = 0.39 and 0.45 respectively) there is evidence to suggest that these are comparable with the reported stability of more established personality traits in cattle. For example, one longitudinal study in cattle reported that exploration and boldness, as inferred from two behavioural tests taken 6 months apart, had correlations of 0.33 and 0.49 respectively indicating temporal stability^13^. Our reported correlation between contexts is also in line with the magnitude of correlation which has been used to support trait continuity in children, with one meta-analysis of longitudinal studies of established personality traits indicating correlations of 0.41 within childhood^30^. In humans there is evidence to suggest individual continuity in eating behaviours (such as eating speed) from the age of four years. This could indicate that between-individual differences in eating behaviour may also remain relatively stable in non-human animals^31^. If between individual differences in calf feeding behaviour persist into later life, these differences could inform individual management strategies in older cattle. One recent study in cattle reported that boldness and exploration personality traits had poor stability across puberty (i.e., from pre- and post-weaning to lactation) but were consistent from pre- and post-weaning to puberty and from puberty to lactation indicating long term consistency within these developmental periods^32^. Further research is needed to explore the longer-term stability of between individual differences in calf feeding behaviour. It is worth noting that our study explored between individual differences over two different contexts which mainly differed in terms of their group size and access to the feeder. Further research is needed to test if changes to the milk feeding regime (e.g., milk allowance, meal size, meal timings etc.) as well as if reductions in feed allowance during weaning affect the relative stability of the between individual differences in feeding behaviour reported here.

Our results show that calves that drank more quickly and those that had higher meal frequency gained more weight. This result indicates that calves that are situated on the higher meal frequency and faster feeding rate end of the proposed personality trait have higher growth rates. Indeed, there is evidence that personality traits are related to production in livestock^12,33,34^. For example, several studies have reported that increased ‘fearfulness’ or increased ‘reactivity to handling’ is significantly associated with reduced weight gain in beef cattle and reduced milk production in dairy cattle^35^. Exploration, as measured by behavioural tests, has been associated with performance with one study in calves reporting that more exploratory individuals gained more weight^12^. However, a similar study in calves reported no difference^36^. More broadly, our results are in line with the ‘pace-of-life syndrome’ which predicts that individuals on the ‘faster’ end of the fast/slow continuum tend to have higher metabolism and grow more quickly^37^. The pace of life hypothesis draws associations between range of physiological characteristics and personality traits. Future research could explore whether calves at the faster and slower ends of the feeding personality trait continuum differ in terms physiological processes, such as metabolism or appetite regulation. Future research is also needed to investigate if these ‘faster’ calves may also suffer the trade-offs predicted by the ‘pace of life syndrome’ such as later reproduction and increased susceptibility to infectious disease.

Despite the marked differences between the pair housing and the group housing contexts, repeatability estimates for feeding rate and meal frequency remained moderate. This result indicates that, within each context, substantial between-individual variation exists. Between-individual variation in feeding behaviours has been reported in cattle^38–40^, as well as our previous work in calves^23^. Indeed, there is some evidence to suggest that between individual differences in feeding behaviours in cattle have a genetic component with heritability estimates of 0.44 for feeding rate and 0.51 for number of visits to the feedbunk^41^. The repeatability estimates in this study build upon previous results since this is the first to study to estimate repeatability in such young animals (mean age of 15 days in the pair housing context) indicating that between individual differences in feeding behaviour exist from a young age. Furthermore, since between individual differences persisted despite the substantial changes in context (from pair to group housing) our results suggest that these differences may be relatively robust to contextual change. Our results also show that within each context, a positive and significant correlation between calves’ feeding rate and meal frequency exists. This relationship was present in both contexts suggesting that it too is relatively robust to contextual change. Feeding rate and meal frequency are mathematically distinct (i.e., the calculation of feeding rate is independent to that of meal frequency) indicating that this relationship is not the result of a mathematical artefact. Instead, this correlation could result from an underlying personality trait responsible for driving the co-expression of these behaviours. This is supported by some evidence in growing beef cattle which reported that feeding rate and meal frequency were positively though weakly correlated (r = 0.20)^38^, indicating that this relationship may persist into later life. It is worth highlighting that meal frequency in our study was a combination of entitled and non-entitled meals (where the calf visits a feeder but is not allocated a proportion). It has been suggested that non-entitled visits to the feeder may have an exploratory component (i.e., the calf is testing if any milk is available)^42^. Further research is needed to determine how between-individual differences in calf feeding behaviour at an automatic milk feeder relate to feeding behaviours post-weaning.

One limitation of our study is that despite the differences reported meeting the definition for a personality trait, it is not yet possible to position this proposed personality trait in relation to those already established in the literature. However, there is some evidence to suggest that the proposed trait could be linked to ‘exploration’ and/or ‘boldness’. For example, in one study, exploratory/active calves, as inferred from behavioural tests, had more visits to feeder and greater liveweight gain^12^. Another similar study reported that more exploratory calves tended to drink more quickly^36^. Furthermore, bolder, and more exploratory individuals tend to grow more quickly and are therefore situated on that faster end of the ‘pace of life syndrome’ slow/fast axis^7^. Since calves on the faster end of the trait also gained more weight, this could also indicate a relationship with our proposed personality trait and boldness/exploration. Whilst future research could focus on the correlating between individual differences in measured behaviours with the results of suites of behavioural tests to infer personality, it is worth highlighting that these tests also have limitations^43^. Criticisms include the fact the individuals are generally tested alone therefore their behaviour therefore may not generalise well to a social or group situation^44^. A different avenue to improving the interpretability of the proposed trait could be to use sensors to monitor other behaviours (e.g., location, activity etc.) alongside feeding behaviour. Data driven approaches, as we present here, could assist in uncovering other personality traits (e.g., activity, exploration, sociality, aggressiveness etc.), enabling our proposed feeding personality trait to be interpreted within the context of a multi-dimensional personality framework.

## Supplementary Information

Supplementary Information.

## Data Availability

Data is available from the corresponding author upon reasonable request.
