# Supplementary material for: Indication of a personality trait in dairy calves and its link to weight gain through automatically collected feeding behaviours
Source: Sci Rep. 2022 Nov 12;12:19425. doi: 10.1038/s41598-022-24076-x (PMC9653382; doi:10.1038/s41598-022-24076-x)
Supplement: Supplementary file 1 — Supplementary Information. [file 41598_2022_24076_MOESM1_ESM.docx]

**Supplementary Material**

**Meal criterion**

The meal criterion corresponds to the maximum time interval between two visits to the feeder by the same calf for those visits to be grouped into the same meal^1,2^. Thus, those visits that are clustered in time are counted as the same meal. To determine the meal criterion, we calculated the interval in seconds between consecutive visits to the feeder for each calf. We plotted its log10-transformed distribution which revealed three distributions that have intersections of approximately 100 and 1600 seconds. We chose the shorter 100s interval for our meal criterion since we are interested in the quantifying returns to the feeder that were separated by less than 1600s. Figure 1 shows plots the distribution of the log10 transformed intervals between visits.

**
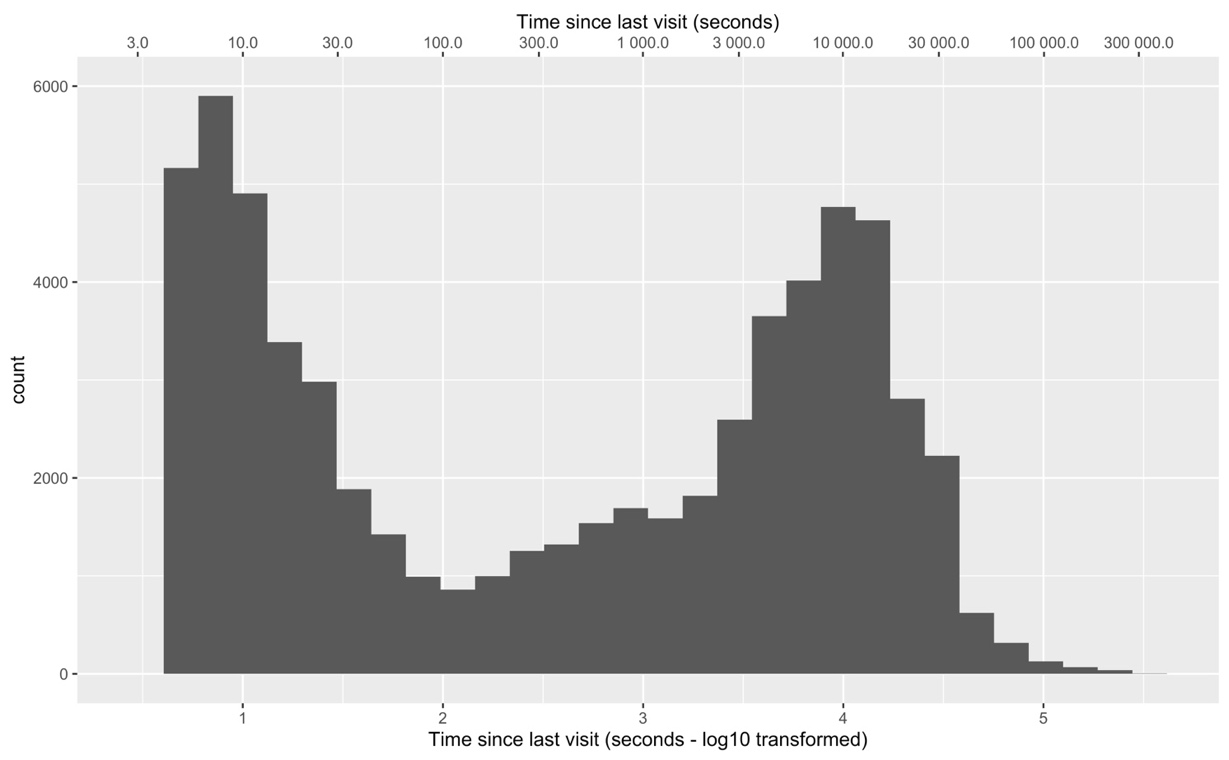
**

**Fig 1. Log 10 transformed time interval between consecutive visits**

Three distributions with intersections of ~10^2 (1000 seconds) and ~10^3.2 (1585 seconds) can be seen in the figure.

**Weight data pre-processing**

Weight data was collected by a partial weight scale that was situated in front of the milk feeding station. Each time the calf visits the feeder a weight is recorded by the computerised feeder. These data were downloaded and appended alongside the feeding data.

After filtering null values, weight values were visually inspected which revealed outlier observations that occurred alongside observations that were consistent with an expected daily liveweight gain of between 0.7 – 1.2kg (see figure 2.) Outlier observations were assumed to occur due to misplacement of the calves’ front hooves on the partial scale (e.g., the calf places one hoof rather than both hooves on the weight scale) resulting in a drop in weight when compared with previous recordings. Erroneous measurements greater than the calf’s true bodyweight were assumed less likely to occur since they would require the calf to assert downward pressure on the partial weight scale. To process the weight data, we fitted a quantile regression model (tau = 0.9) of weight on day number for each individual calf^3^. Quantile regression allows us to assign greater influence to those observations in the quantile of interest (here the top 10%). To assess the deviation of each observation, we calculated the residual mean and standard deviation. We excluded those observations where the residual estimate was less than residual mean or where its absolute value was greater than the residual standard deviation. Secondly, for each calf we ran a linear regression of weight on day number and excluded those observation where the cook’s standard deviation exceeded 0.2 or where the absolute value of the residual exceeded the standard deviation for all residuals.

**
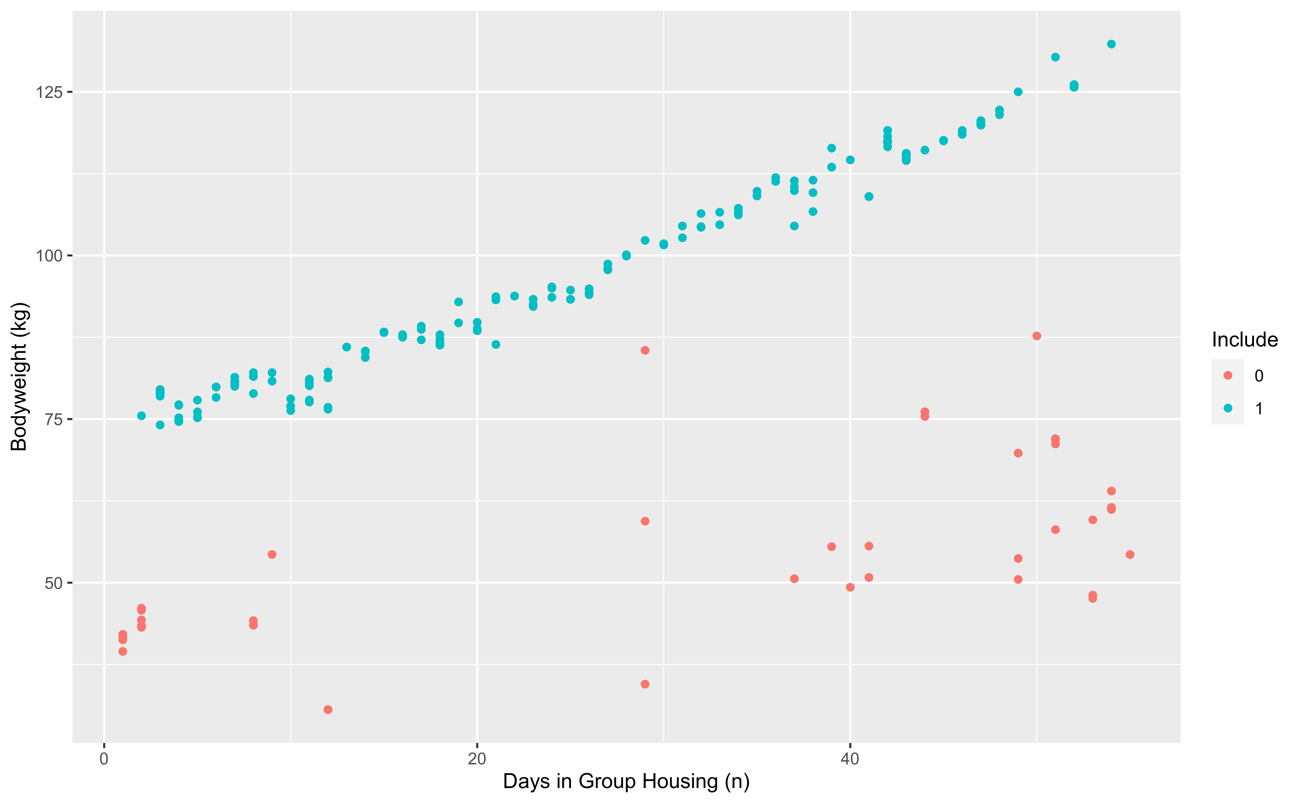
**

**Fig 2. Example of raw data collected by the partial weight scale for one calf over the group housing period. The red observations correspond to those that were excluded after the pre-processing whereas the blue observations correspond to those that were included.**

**Weight estimation**

For each calf, we fitted a linear regression of bodyweight on day number. Weight at the start of the group housing period was estimated by extracting the intercept from the linear model. For each calf, weight at 70 days old was calculated from the model prediction for the corresponding day number.

**References**

1. Tolkamp, B. J. & Kyriazakis, I. To split behaviour into bouts, log-transform the intervals. *Anim Behav* **57**, 807–817 (1999).

2. Tolkamp, B. J., Allcroft, D. J., Austin, E. J., Nielsen, B. L. & Kyriazakis, I. Satiety Splits Feeding Behaviour into Bouts. *Journal of Theoretical Biology* **194**, (1998).

3. Koenker, R. & Bassett, G. Regression Quantiles. *Econometrica* **46**, 33 (1978).
